# The Action of Water on Lead

**Published:** 1891-06

**Authors:** 


*The Action of Water on Lead.* By JOHN HENRY GARRETT, M.D. London: H. K. LEWIS. 1891.

The constant occurrence of lead-poisoning, to an extent productive of considerable injury to the public health, in certain districts where the drinking-water exerts a continuous action on the lead service-pipes, has of late aroused considerable interest; and many practical chemists have directed attention to the exact nature of the action, and to the determination of the reasons why some waters—notably the moorland surface-waters of Yorkshire, which are otherwise of excellent quality—possess this lead-dissolving power in so remarkable a degree. At the present time, when the attention of the Government has been directed to this important question, Dr. Garrett's careful experiments and judicious interpretation of results may be commended as well worthy of study, and as throwing a not inconsiderable light upon this somewhat obscure problem.

